# Draft genome sequences of three *Carnobacterium maltaromaticum* isolates from two low pH bogs in Denmark

**DOI:** 10.1128/mra.00969-23

**Published:** 2023-12-01

**Authors:** Taya Tang, Jørgen J. Leisner

**Affiliations:** 1 Department of Veterinary and Animal Sciences, Faculty of Health and Medical Sciences University of Copenhagen, Frederiksberg, Denmark; DOE Joint Genome Institute, Berkeley, California, USA

**Keywords:** carnobacteria, freshwater microbiology, lactic acid bacteria

## Abstract

*Carnobacterium maltaromaticum* is a lactic acid bacterium that is widely distributed in the environment, including freshwater. Here, we report the draft genome sequences of three *C. maltaromaticum* isolates from low pH Danish bogs, using the Illumina MiSeq platform.

## ANNOUNCEMENT

The lactic acid bacterium *Carnobacterium maltaromaticum* is widely distributed in Danish freshwater environments during the winter season but underrepresented in bogs and poor fens (<pH 5.15) (1). Here, we show that *C. maltaromaticum* could be readily isolated from such habitats when culture-dependent sampling was done during the summer season and by using an enrichment procedure including an increased volume of sample relative to enrichment medium.

Ten milliliters of water samples were obtained on 30 May 2023, from two sites (Table 1) (1). One or three milliliters of samples were mixed 1:1 with all purpose Tween (APT) broth (Difco, Sparks, MD, USA) and incubated anaerobically at 15°C for 3 d before plating on de Man, Rogosa, Sharpe (MRS) agar (Oxoid, Thermo Fisher Scientific, Waltham, USA) anaerobically at 15°C for 3–5 d. Single colonies of Gram-positive and catalase-negative isolates (s2-1 from site 1; s3-1 and s6-2 from site 2) were cultivated in 3-mL APT broth under anaerobic and static conditions at 15°C for 3 d. Bacterial cells were harvested by centrifugation, and DNA extraction was performed using a DNeasy Blood and Tissue Kit, according to the manufacturer’s instructions (Qiagen GmbH, Hilden, Germany). DNA concentrations were measured by NanoDrop One Spectrophotometer (Thermo Fisher Scientific). Library preparation was done using the Nextera DNA Flex Library Prep Kit (Illumina, San Diego, USA). Sequencing on an Illumina MiSeq platform was performed as described by Li et al. (2), generating paired-end reads of 2 × 300 bp. An initial quality control involved FastQC v.0.11.9 (3) and trimming low-quality bases (*Q* <20) and adapter sequences with Cutadapt v.4.4 (4). Trimmed reads were assembled using SPAdes v.3.13.1 (5) with assembly quality assessed by QUAST 5.2.0 (6). Genome annotation was performed with Prokka v. 1.13 (7). Within the assembled contigs, the 16S rRNA gene regions were identified using Barrnap v.0.9 (8). The corresponding sequences were extracted using Bedtools v2.30.0 (9). Identification of the isolates was performed by comparing their 16S rRNA gene sequences via BLAST against the NCBI 16S rRNA database. A phylogenetic tree based on single-nucleotide polymorphism (SNP) variants in the whole core genomes was constructed using sequences of the 3 isolates from this study, 28 freshwater isolates of *C. maltaromaticum* from our previous study (BioProject: PRJNA990564, BioSample: SAMN36274541 to SAMN36274568) (1) and 19 published genome sequences of *C. maltaromaticum*, *Carnobacterium gallinarum,* and *Carnobacterium* sp. retrieved from the NCBI database. SNPs were identified by mapping reads against the reference *C. maltaromaticum* ATCC 35586 genome (GCF_000238575.1) via CSI Phylogeny v1.4 (10) and visualized in ChiPlot (11). Finally, we probed the presence of genes encoding bacteriocins by AntiSMASH 6.0 (12) and BAGEL 4 (13). All bioinformatic tools were run with default parameters.

**TABLE 1:** Sampling location description and genome characteristics for the three *C. maltaromaticum* isolates

Variable	s2-1	s3-1	s6-2
Location	Site 1	Site 2	Site 2
Type	Forest bog	Forest bog	Forest bog
pH	4.35	4.40	4.30
Geographic coordinates	55°45*'*12.0*"*N 12°30*'*17.3*"*E	55°49*'*35.0*"*N 12°33*'*40.5*"*E	55°49*'*34.8*"*N 12°33*'*40.4*"*E
Genome characteristics			
Assembly size (bp)	3,728,548	3,498,272	3,930,617
N50 (bp)	181,003	188,278	246,871
L50 (bp)	7	6	8
GC (%)	34.10	34.34	34.14
Number of contigs	73	47	76
Number of CDS	3,372	3,141	3,566
Number of tRNAs	57	63	62
Number of rRNAs	8	7	12
Number of sequences	645,632	449,296	222,660
Total number of reads (bp)	193,689,600	134,788,800	66,798,000
Genome coverage	55×	38×	19×
Bacteriocins^*a*^	BM1	BM1	None
BioProject accession	PRJNA990564	PRJNA990564	PRJNA990564
BioSample accession	SAMN37661217	SAMN37661256	SAMN37661257
SRA accession for raw reads	SRR26265303	SRR26265302	SRR26265301
GenBank accession	JAWLUX000000000	JAWLUY000000000	JAWLUZ000000000

^a^ Presence of gene encoding the structure of an unmodified bacteriocin.

**Fig 1:**
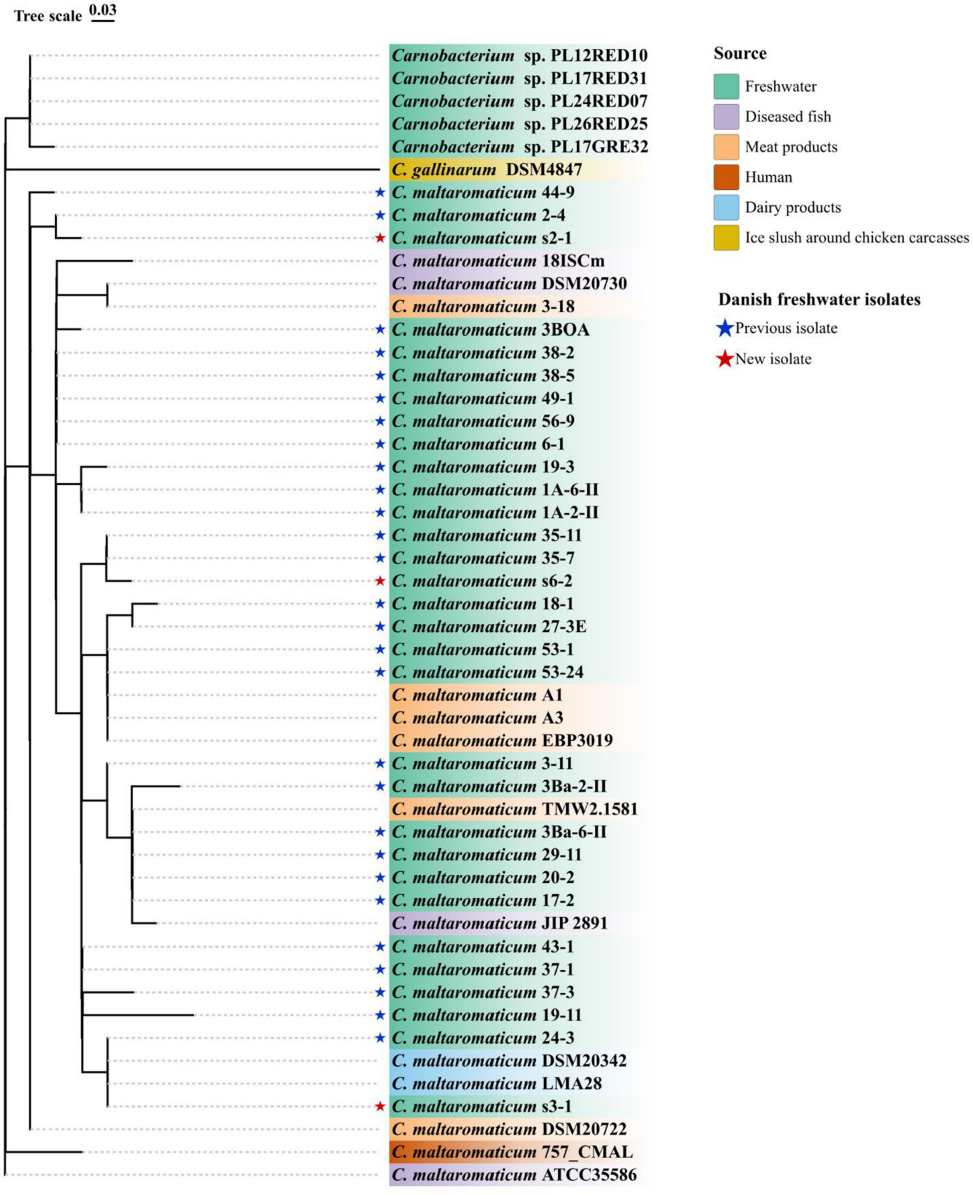
A maximum-likelihood phylogenetic tree based on SNP variants in the whole core genomes of the *Carnobacterium* spp. strains. *C. maltaromaticum* ATCC 35586 was used as the reference genome to map and screen the SNPs.

Sequence statistics of the *C. maltaromaticum* genomes are summarized in Table 1, and a phylogenetic tree shows the close relatedness of the three isolates from this study to other *C. maltaromaticum* strains (Fig. 1). Genes encoding carnobacteriocin BM1 and the corresponding immunity protein were found in the s2-1 and s3-1 genomes. No genes encoding structures of other bacteriocins were detected.

## Data Availability

All raw sequence reads have been deposited at the NCBI under BioProject with accession number PRJNA990564, BioSamples with accession numbers SAMN37661217, SAMN37661256, and SAMN37661257, and SRA with accession numbers SRR26265301, SRR26265302, and SRR26265303. Whole-genome sequences have been deposited at GenBank under the accession numbers JAWLUX000000000, JAWLUY000000000, and JAWLUZ000000000. The accession numbers for the individual isolates are given in Table 1.

## References

[1] Tang T , Martinenghi LD , Hounmanou YMG , Leisner JJ . 2023. Distribution and ecology of the generalist lactic acid bacterium Carnobacterium maltaromaticum in different freshwater habitats: metabolic and antagonistic abilities. Environ Microbiol. doi:10.1111/1462-2920.16508 37750577

[2] Li H , Ramia NE , Borges F , Revol-Junelles A-M , Vogensen FK , Leisner JJ . 2021. Identification of potential citrate metabolism pathways in Carnobacterium maltaromaticum. Microorganisms 9:2169. doi:10.3390/microorganisms9102169 34683489 PMC8537297

[3] Andrews S . 2010. Fastqc: a quality control tool for high throughput sequence data. Babraham Bioinformatics, Babraham Institute, Cambridge, United Kingdom.

[4] Martin M . 2011. Cutadapt removes adapter sequences from high-throughput sequencing reads. EMBnet j 17:10. doi:10.14806/ej.17.1.200

[5] Prjibelski A , Antipov D , Meleshko D , Lapidus A , Korobeynikov A . 2020. Using spades de novo assembler. Curr Protoc Bioinformatics 70:e102. doi:10.1002/cpbi.102 32559359

[6] Gurevich A , Saveliev V , Vyahhi N , Tesler G . 2013. QUAST: quality assessment tool for genome assemblies. Bioinformatics 29:1072–1075. doi:10.1093/bioinformatics/btt086 23422339 PMC3624806

[7] Seemann T . 2014. Prokka: rapid prokaryotic genome annotation. Bioinformatics 30:2068–2069. doi:10.1093/bioinformatics/btu153 24642063

[8] Seemann T . 2021. Barrnap 0.9: rapid ribosomal RNA prediction. https://github.com/tseemann/barrnap/09

[9] Quinlan AR , Hall IM . 2010. Bedtools: a flexible suite of utilities for comparing genomic features. Bioinformatics 26:841–842. doi:10.1093/bioinformatics/btq033 20110278 PMC2832824

[10] Kaas RS , Leekitcharoenphon P , Aarestrup FM , Lund O . 2014. Solving the problem of comparing whole bacterial genomes across different sequencing platforms. PLoS One 9:e104984. doi:10.1371/journal.pone.0104984 25110940 PMC4128722

[11] Xie J , Chen Y , Cai G , Cai R , Hu Z , Wang H . 2023. Tree visualization by one table (tvBOT): a web application for visualizing, modifying and annotating phylogenetic trees. Nucleic Acids Res 51:W587–W592. doi:10.1093/nar/gkad359 37144476 PMC10320113

[12] Blin K , Shaw S , Kloosterman AM , Charlop-Powers Z , van Wezel GP , Medema MH , Weber T . 2021. antiSMASH 6.0: improving cluster detection and comparison capabilities. Nucleic Acids Res 49:W29–W35. doi:10.1093/nar/gkab335 33978755 PMC8262755

[13] van Heel AJ , de Jong A , Song C , Viel JH , Kok J , Kuipers OP . 2018. Bagel4: a user-friendly web server to thoroughly mine RiPPs and bacteriocins. Nucleic Acids Res 46:W278–W281. doi:10.1093/nar/gky383 29788290 PMC6030817

